# Accouchement différé d´une grossesse multiple: à propos d´un cas et revue de la littérature

**DOI:** 10.11604/pamj.2020.36.373.19797

**Published:** 2020-08-31

**Authors:** Mehdi El Hassani, Jihad Drissi, Saad Benali, Abdellah Baba Habib, Jaouad Kouach, Driss Moussaoui

**Affiliations:** 1 Service de Gynécologie-Obstétrique, Hôpital Militaire d´Instruction Mohamed V, Rabat, Maroc

**Keywords:** Grossesse multiple, accouchement différé, prématurité, Multiple pregnancy, delayed delivery, prematurity

## Abstract

L´accouchement gémellaire différé a pour but de prolonger la vie intra-utérine du ou des fœtus restants après l´expulsion prématurée du premier jumeau au deuxième trimestre de la grossesse, en vue d´atteindre un âge gestationnel compatible avec la survie néonatale pour le ou les fœtus restants. L´objectif de ce travail est de mettre en exergue le bénéfice et les indications de cette pratique obstétricales, et ce, à travers la description d´un cas clinique d´un accouchement gémellaire différé chez une patiente porteuse d´une grossesse triple, et chez qui le délai entre l´expulsion du premier jumeau et l´accouchement du troisième jumeau était de 10 semaines.

## Abstract (en)

Delayed delivery is designed to allow the remaining fetus(es) to develop after premature expulsion of the first twin in the second trimester of pregnancy. This decision is aimed to allow the remaining fetus(es) to reach full fetal growth. We here report a clinical case of delayed delivery in a patient with triple pregnancy in whom the time between the expulsion of the first twin and the birth of the third twin was 10 weeks. The purpose of this study was to highlight the benefit and indications for delayed delivery.

## Introduction

Les grossesses gémellaires sont devenues un enjeu majeur en obstétrique puisque leur nombre est en hausse du fait du recul de l´âge maternel et du recours aux techniques d´assistance médicale à la procréation. Ces grossesses sont associées à une morbidité materno-fœtale plus élevée qu´en cas de grossesse singlette et posent des problèmes à tous les stades: suivi, accouchement et post-partum. En effet, la menace de fausse couche tardive avec rupture prématurée des membranes avant 28 semaines d´aménorrhée complique 1,37% des grossesses multiples avec une mortalité périnatale qui varie entre 47 et 120% pour les grossesses gémellaires et 93 et 203% dans les grossesses triples [1]. Ainsi, l´accouchement gémellaire différé a pour but de prolonger la vie intra-utérine du ou des fœtus restants après l´expulsion prématurée du premier jumeau en vue d´atteindre un âge gestationnel compatible avec la survie néonatale pour le ou les fœtus restants [1, 2]. Nous rapportons le cas d´un accouchement gémellaire différé chez une patiente porteuse d´une grossesse triple, chez qui le délai entre l´expulsion du premier jumeau et l´accouchement du troisième jumeau était de 10 semaines. L´objectif de ce travail est de mettre en exergue le bénéfice et les indications de cette pratique obstétricale.

## Patient et observation

Il s'agit de Mme O. E. Agée de 42 ans, ayant eu une fécondation in vitro (FIV), après une infertilité secondaire de 8 ans et plusieurs échecs en procréation médicalement assistée (PMA). Dans ses antécédents on note une césarienne, une grossesse extra-utérine traitée par salpingectomie et une myomectomie. La grossesse actuelle est une grossesse triple, cerclée a 12 SA, suivie pour menace d´avortement depuis 14 SA dans notre formation. Sa grossesse s'était compliquée d´une rupture de la poche des eaux d´un des triplets le plus bas situé à 16 SA, la patiente était apyrétique avec des contractions utérines et hydrorrhée faite de liquide clair, l'échographie avait montré une grossesse triple évolutive bichochorial triamniotique (j1 et j2 même placenta) sans anomalies visibles et dont la biométrie correspond à l´âge gestationnel (AGT) Avec un liquide amniotique en quantité diminuée pour le premier jumeau. Le bilan infectieux était négatif et la patiente a été mise sous traitement progestatif et antibioprophylaxie avec surveillance de la température et la CRP vue le risque infectieux (explications données à la patiente sur le pronostic réservé quant à l´issue de la grossesse). L´évolution était favorable et la patiente était sortie sous traitement avec surveillance de la température et de la CRP (restées normales).

Elle est réadmise à 20 SA pour reprise de la menace d´avortement tardif, l´examen avait objectivé des contractions utérines sans signe d´appel infectieux le col était fermé (cerclée) et propre. L´échographie avait montré une grossesse triple évolutive avec biométrie correspondant à l´AGT et un liquide amniotique en quantité réduite pour le J1. Sur le plan biologique la CRP est revenue à 30 mg/l avec des globules blancs normaux, l´ECBU était stérile. La patiente fut tocolysée par Nicardipine en IV avec bi-antibiothérapie à base de Céphalosporine de troisième génération associée à la Gentamycine, l´évolution était favorable avec maintien d´un état d´apyrexie, amendement des contractions utérines et normalisation de la CRP. A 21 SA, le premier jumeau décède in utéro puis fut expulsé spontanément à 22 SA, fil de cerclage exclue. Devant l´absence de chorioamniotite avec apyrexie et CRP stable autour de 20 mg/l, l´arrêt des contractions utérines, l´absence de saignement et la bonne évolution des deux fœtus restants, l´indication d´un accouchement différé a été portée en concertation avec le couple. Une ligature-section du cordon à ras du col était alors faite. La tocolyse était reprise par Nicardipine en IV et antibiothérapie avec surveillance régulière des paramètres cliniques et biologiques.

A 23 SA, l´évolution était marquée encore une fois par la rupture de la poche de J2 suite à la reprise des contractions utérines et expulsion spontanée d´un fœtus vivant de sexe féminin pesant 530g et décédé à 30 min de vie. Vu la disparition de l´activité utérine, l´absence de décollement placentaire et d´hémorragie, l´absence de signe d´appel infectieux clinico-biologique (CRP, NFS, PV et ECBU), une ligature section du cordon à ras du col a été réalisée avec maintien de la tocolyse et de l´antibiothérapie prophylactique. Vu l'impossibilité de transfert in utéro dans une maternité niveau III pour prise en charge, vu le contexte de cette grossesse (patiente de 42 ans avec stérilité et FIV difficile), et sur demande du couple (bien conscient des risques). Nous décidons de continuer la même prise en charge (tocolyse IV, antibiothérapie avec des fenêtres thérapeutiques, repos, hydratation et corticothérapie à partir de 28 SA et bilans infectieux réguliers ainsi que des échographies) en vue d´un accouchement différé permettant au fœtus restant d´atteindre la viabilité ce qui nous a permis de prolonger la grossesse jusqu'à 30 SA. A 30 SA, la patiente a présenté des métrorragies de moyenne abondance suite à une reprise des contractions utérines avec décollement prématuré du placenta, d´où la décision de césarienne en urgence permettant l´extraction d´un nouveau-né de sexe féminin Apgar à 10/10 pesant 1230g pris en charge en néonatalogie. Les suites opératoires étaient simples pour la patiente qui est sortie à J5 du post partum. Quant au nouveau-né il a présenté une infection traitée sans complications et la petite fille a été remise à sa mère après un séjour de quatre semaines en néonatalogie, le contrôle pédiatrique à 1, 3, 6 et 12 mois était normal.

## Discussion

Depuis le premier cas décrit en 1880 par Curson, plus de 70 cas d´accouchements gémellaires différés ont été rapportés dans la littérature avec une durée d´intervalle entre l´expulsion du premier et du dernier jumeau de 4 à 143 jours [1, 3]. Plusieurs définitions de l´accouchement différé ont été proposées: Depaul [2, 4] parle d´un accouchement en deux temps; Arias [2, 5] le définit comme une accouchement gémellaire pour lequel le premier jumeau est sorti au deuxième trimestre et le second jumeau au troisième trimestre; Cristinelli [2, 6] parle d´un accouchement en plusieurs temps avec expulsion du premier jumeau au deuxième ou au troisième trimestre et un prolongement de la grossesse pour obtenir l´accouchement du ou des fœtus restants le plus proche du terme. La notion du terme a ici toute sa valeur. En effet, les expulsions du premier jumeau au premier trimestre ne sont pas intégrées dans la définition. Par ailleurs, les progrès de la réanimation néonatale dans la prise en charge des nouveau-nés de plus de 28 semaines d´aménorrhée soulèvent la question de la balance bénéfice-risque de l´application de cette pratique obstétricale au troisième trimestre de la grossesse [2, 7, 8]. Ceci dit, l´accouchement gémellaire différé a pour but de prolonger la vie intra-utérine du ou des fœtus restants après expulsion du premier jumeau au deuxième trimestre [2]. Dans notre observation, l´expulsion de J1 est survenue à 18 SA alors que l´accouchement du troisième jumeau ne s´est produit qu´à 30 SA. Seules les grossesses multichoriales multiamniotiques sont éligibles à cette pratique qui ne peut s´envisager que dans les rares cas où l´activité utérine cesse de façon durable après l´expulsion du premier jumeau [1].

En outre, il est impératif d´exclure toutes les contre-indications avant de discuter la possibilité d´un accouchement différé. En effet, l´absence de saignement abondant après expulsion de J1, l´absence de signes évocateurs d´HRP, de SFA ou de chorioamniotite sont des préalables nécessaires à vérifier avant l´acceptation de l´application de cette pratique obstétricale [2]. Tous ces éléments ont bien évidemment été vérifiés dans notre cas clinique. Une fois l´indication portée un certain nombre de gestes doivent être réalisés: la ligature du cordon au ras du col, réalisée dans notre cas sous valves, elle limite le risque infectieux; la tocolyse peut être proposée comme attitude préventive [2, 6], bien que certains auteurs ne la préconisent pas après arrêt spontané de l´activité utérine; la réapparition des contractions utérines serait le témoin d´une infection sous-jacente notamment ovulaire pour laquelle la prolongation de la grossesse ne ferait qu´augmenter les risque de morbidité materno-fœtale comme cela a été souligné dans l´étude de Porreco portant sur 24 cas mis sous tocolyse préventive et dans laquelle 7 cas d´endométrite aiguë et un cas de phlébite pelvienne septique ont été recensés [2, 9]. Dans notre cas une tocolyse prophylactique par Nicardipine a été instaurée.

La corticothérapie est quasi-systématique devant les risques de la grande prématurité [2]. Le cerclage après expulsion de J1 est utilisé dans la littérature dan 46% des cas, mais le débat reste d´actualité vu ses risques inhérents (rupture des membranes et risque infectieux non négligeable). Par ailleurs, certains auteurs ont prouvé que la pratique d´un cerclage après expulsion de J1 permet de prolonger l´intervalle de naissance entre les jumeaux alors que l´existence d´un cerclage préalable à l´expulsion de J1 était associée un intervalle plus court; il n´est cependant pas recommandé de recercler ces patientes [2]. L´antibioprophylaxie est systématique puisque l´ouverture de l´œuf est associée à un risque infectieux accru par contamination ascendante. Cristennelli propose une durée d´antibiothérapie de 7 à 10 jours à reprendre en cas de réapparition de signes infectieux [2, 6]. Pour certains auteurs cette antibiothérapie devrait être adaptée au germe retrouvé sur les prélèvements vaginaux réalisés tous les 8 jours. En effet, deux cas de colite pseudo-membraneuse à *Clostridium* ont été décrits dans la littérature suite à une antibioprophylaxie [1]. Chez notre patiente les antibiotiques ont été administrés avec fenêtres thérapeutiques.

## Conclusion

L´accouchement gémellaire différé est une pratique obstétricale en progression vu l´augmentation de la prévalence des grossesses multiples. Elle est réalisée dans l´optique de prolonger la vie intra-utérine du ou des jumeaux restants après expulsion du premier fœtus et ceux jusqu´à atteindre un âge gestationnel compatible avec un bon pronostic de survie néonatale. Cependant, cette attitude n´est pas dénuée de risques de complications materno-fœtales d´où l´intérêt de savoir en poser l´indication judicieuse.
